# Editorial: Roles of Liver Sinusoidal Endothelial Cells in Liver Homeostasis and Disease

**DOI:** 10.3389/fphys.2022.869473

**Published:** 2022-03-08

**Authors:** Patricia F. Lalor, Thomas Huser, Leo A. van Grunsven

**Affiliations:** ^1^Centre for Liver and Gastroenterology Research and National Institute for Health Research (NIHR) Birmingham Biomedical Research Centre, Institute of Immunology and Immunotherapy, University of Birmingham, Birmingham, United Kingdom; ^2^Biomolecular Photonics Research Group, Faculty of Physics, Bielefeld University, Bielefeld, Germany; ^3^Liver Cell Biology Research Group, Vrije Universiteit Brussel, Brussel, Belgium

**Keywords:** sinusoidal endothelial cell, liver, chronic liver damage, scavenger receptor, super-resolution imaging, gene expression

As a consequence of rising global rates of metabolic disease and alcohol-related injury, the research community has a heightened interest in understanding the mechanisms which underlie hepatic inflammation and fibrogenesis. Specific immune cell populations and hepatic stellate cells have obvious significance in this regard, but one underestimated cell population deserves greater prominence. Hepatic sinusoidal endothelial cells are vital to metabolic and functional homeostasis, and changes in their phenotype as a consequence of injury or aging have an impact on disease pathophysiology. In this article series our authors have highlighted both novel mechanistic approaches, and novel features of liver sinusoidal endothelial cells (LSEC) which have great potential for preclinical development.

Bhandari et al. review the phagocytic and clearance roles of LSEC to consider their function as part of the hepatic reticuloendothelial system. Scavenger functions protect liver parenchyma from exposure to noxious substances. Hence scavenger receptors contribute to clearance of viruses, macromolecules, and nanosized pharmaceuticals and also contribute to immune defense by supporting immune cell recruitment. In addition to their consideration of key receptors involved in clearance, the authors also comment on anatomical features of LSEC such as their fenestrations, large surface area and abundant endocytic machinery which support the fast and efficient clearance roles of these cells. There is also consideration of the evolutionary conservation of scavenger endothelial functions to emphasize the conserved importance of the clearance capacities of such cells in vertebrate species. In another contribution, Pandey et al. provide more detail on the structure of hepatic sinusoidal endothelial cells and revisit key scavenger receptors expressed by LSEC to highlight the importance of the clearance capacity of these cells. Here, the authors highlight the diversity of possible ligands cleared by LSEC. They also consider both homeostatic clearance of endogenous lipids and degradation products, and the uptake of lipopolysaccharides (LPS), pathogen associated molecules and oxidized lipids in a diseased context. Changes in receptor expression profiles which occur in chronic disease or cancer in both rodents and humans are also reviewed. Furthermore, they also provide an interesting perspective on LSEC adhesion molecules involved in the regulation of liver inflammation and cell recruitment from the bloodstream, which are important mechanisms for recruiting progenitor cells in the context of disease. Tripathi et al. consider the angiogenic role of circulating bone marrow-derived endothelial progenitor cells in cirrhosis. They show that CD34+VEGFR2+ cells can be detected in human blood and are increased in cirrhosis. These cells express endothelial-like characteristics when cultured *in vitro* and infiltrate the liver after intravenous administration in rats. There appears to be a change in phenotype of these cells in the context of cirrhosis such that cells from cirrhotic patients seem to enhance rodent fibrotic injury and portal hypertension when compared to cells from healthy individuals. This suggests not only that dedifferentiation of endothelial precursor cells occurs in cirrhosis, but also that normal repair mechanisms which maintain hepatic vascular homeostasis in health may be compromised in chronic injury. Thus, diseased endothelial precursors and local de-differentiated LSEC contribute to fibrogenesis in disease.

Wilkinson et al. turn their attention specifically to LSEC in the context of cancer and chronic disease. Here, they explain the mechanistic basis by which capillarization of LSEC contributes to portal hypertension, altered thrombogenesis, chronic inflammation and perpetuation of fibrogenesis. By focusing on the influence of the microenvironment around LSEC with respect to their function, the authors consider underexplored metabolic and angiogenic aspects of LSEC function, alongside their role in secondary metastasis to highlight novel approaches for cancer therapies. Candidate molecules such as VAP-1 and SCARF-1, and novel approaches using targeted nanoparticles and miRNAs are considered as potential therapeutic tools to manage the increasing burden of liver cancer. In another contribution, a mechanistic explanation for maintenance of LSEC phenotype is provided by Koch et al., who used mice with constitutive activation of β-catenin in endothelial cells. They confirm that a basal low level of *Wnt* signaling is crucial for maintenance of mature LSEC phenotype. Gain of function overexpression in LSEC led to a dedifferentiation response and change in lipoprotein transport accompanied by a loss of prototypic LSEC gene expression. This led to adoption of a phenotype similar to vascular endothelial cells in the blood brain barrier where *Wnt* signaling is normally confined. Whilst not all features of capillarization were recreated by this approach, the study provides insight into key angiocrine signatures which impact endothelial phenotype and lipid metabolism. Important endothelial specific gene signatures are also described in a study by Verhulst et al.. The authors harness the power of single cell sequencing datasets to provide a detailed and important bioinformatic analysis of gene expression in healthy and diseased rodent livers and human samples. This allowed identification of key genes which are differentially expressed in diseased human cells and were linked to fibrogenesis and migration. However, cardinal functions such as scavenging appear conserved even in disease since gene signatures for scavenger receptors and viral coreceptors were enriched in both diseased and healthy samples. Acute injury had a profound effect on murine LSEC phenotype with an upregulation of reparatory and inflammatory signaling which persisted for some time. Importantly a comparison of the gene signatures in human and murine LSEC revealed key genes that can differentiate healthy LSEC from diseased cells in both species.

Much of the available evidence on LSEC function and anatomy has historically been derived from rodent studies, so the human study by Kong et al. is significant. The authors address the challenges of imaging anatomical features and LSEC in human liver specimens by providing beautiful evidence highlighting the potential of different advanced microscopy applications. Mesoscale approaches such as lightsheet microscopy and optical projection tomography provide insights into whole tissue, and gross vascular architectural change in disease. Innovative biophysical imaging tools such as coherent Raman and confocal optical microscopy add subcellular detail in a label-free, minimally processed manner thereby preserving the integrity of valuable biopsy specimens. Here, the potential to assess early fibrogenesis and quantify steatosis is particularly impressive. Finally, the power of super-resolution imaging is demonstrated with structured illumination microscopic images of fenestrations in human LSEC in culture. The fenestrations in LSEC are revisited in the review from Szafranska et al., who consider the impact of endogenous agents and pharmacological compounds on liver endothelial cell porosity. This is important as the pharmacokinetics and clearance of molecules by the liver are impacted by transport across the LSEC. Mechanistic regulation of fenestration size and number is also explained, and the authors consider how extending our knowledge of these processes may facilitate development of tools to restore porosity in aging or chronic disease. Macromolecule clearance is also the focus of an article by James et al. who highlight the underappreciated role of LSEC in immune complex clearance. Here, key scavenger receptors again feature but specifically in regard to their roles in the uptake and recycling of therapeutic antibody. This is relevant for intelligent design of new biological drugs. Strategies to maximize efficacy and minimize antibody clearance, and the impact of disease or aging on pharmacokinetics are described. Consideration is also given to how LSEC scavenger receptor mediated clearance may explain some of the off-target side effects of antibody-based therapies.

In combination, the studies presented within this Research Topic demonstrate the diverse and often underexplored functions of the sinusoidal endothelium and provide a great primer for those striving to learn more about these fascinating cells.

## Author Contributions

All authors contributed to the drafting and editing of this document and approved the final version.

## Funding

The authors received funding from the European Union's Horizon 2020 research and innovation program under the Marie Sklodowska-Curie Grant Agreement No. 766181, project DeLIVER. This paper presents independent research supported in part by the National Institute for Health Research (NIHR) Birmingham Biomedical Research Centre at the University Hospitals Birmingham NHS Foundation Trust and the University of Birmingham (BRC-1215-20009).

## Conflict of Interest

The authors declare that the research was conducted in the absence of any commercial or financial relationships that could be construed as a potential conflict of interest.

## Publisher's Note

All claims expressed in this article are solely those of the authors and do not necessarily represent those of their affiliated organizations, or those of the publisher, the editors and the reviewers. Any product that may be evaluated in this article, or claim that may be made by its manufacturer, is not guaranteed or endorsed by the publisher.

